# Inhibition of oxidative stress by apocynin attenuated chronic obstructive pulmonary disease progression and vascular injury by cigarette smoke exposure

**DOI:** 10.1111/bph.16068

**Published:** 2023-03-31

**Authors:** Stanley M. H. Chan, Kurt Brassington, Suleman Abdullah Almerdasi, Aleksandar Dobric, Simone N. De Luca, Madison Coward‐Smith, Hao Wang, Kevin Mou, Alina Akhtar, Rana Abdullah Alateeq, Wei Wang, Huei Jiunn Seow, Stavros Selemidis, Steven Bozinovski, Ross Vlahos

**Affiliations:** ^1^ Centre for Respiratory Science and Health, School of Health and Biomedical Sciences RMIT University Bundoora Victoria 3083 Australia

**Keywords:** apocynin, chronic inflammation, chronic obstructive pulmonary disease, endothelial dysfunction, hyperinflation, NADPH oxidase, platelet activation

## Abstract

**Background and Purpose:**

Cardiovascular disease affects up to half of the patients with chronic obstructive pulmonary disease (COPD), exerting deleterious impact on health outcomes and survivability. Vascular endothelial dysfunction marks the onset of cardiovascular disease. The present study examined the effect of a potent NADPH Oxidase (NOX) inhibitor and free‐radical scavenger, apocynin, on COPD‐related cardiovascular disease.

**Experimental Approach:**

Male BALB/c mice were exposed to either room air (Sham) or cigarette smoke (CS) generated from 9 cigarettes·day^−1^, 5 days a week for up to 24 weeks with or without apocynin treatment (5 mg·kg^−1^·day^−1^, intraperitoneal injection).

**Key Results:**

Eight‐weeks of apocynin treatment reduced airway neutrophil infiltration (by 42%) and completely preserved endothelial function and endothelial nitric oxide synthase (eNOS) availability against the oxidative insults of cigarette smoke exposure. These preservative effects were maintained up until the 24‐week time point. 24‐week of apocynin treatment markedly reduced airway inflammation (reduced infiltration of macrophage, neutrophil and lymphocyte), lung function decline (hyperinflation) and prevented airway collagen deposition by cigarette smoke exposure.

**Conclusion and Implications:**

Limiting NOX activity may slow COPD progression and lower cardiovascular disease risk, particularly when signs of oxidative stress become evident.

**Abbreviations**
COPD: chronic obstructive pulmonary disease
CS: cigarette smoke
PBS: phosphate‐buffered saline

**What is already known**

- Irreversible airflow limitation in COPD is due to narrowing and fibrosis of small airways.
- Cardiovascular disease is a major COPD co‐morbidity that predicts disease morbidity and mortality.

**What does this study add**

- Oxidative stress inhibition lessens airflow limitation and airway fibrosis in a mouse model of COPD.
- Targeting oxidative stress also prevents the onset of endothelial dysfunction and platelet activation in COPD.

**What is the clinical significance**

- Oxidative stress is a ‘treatable trait’ for the respiratory symptoms and cardiovascular disease in COPD.

## 1 INTRODUCTION

Chronic obstructive pulmonary disease (COPD) is an inflammatory respiratory disease characterised by chronic airway obstruction and remodelling, and the destruction of lung tissues that interfere with normal breathing (WHO, 2022). The airway pathologies of COPD are largely irreversible, making COPD a major public health issue, affecting approximately 12% of individuals worldwide and incurring a substantial socio‐economic burden (Adeloye et al., 2015). While in industrialised countries, cigarette smoke (CS) exposure is the primary risk factor for the development of COPD. However, COPD may also be caused by combustion of biomass and air pollution in low‐ to middle‐income countries (Safiri et al., 2022). In addition to pulmonary pathologies, COPD is associated with many co‐morbidities, such as cardiovascular disease, metabolic disorders, neurocognitive impairments and musculoskeletal disorders, which further increase the probability of hospital admission and mortality and thus reducing quality of life. In particular, cardiovascular disease has been demonstrated to be a leading contributor to the morbidity and mortality of COPD, and can be detected independently of traditional risk factors in approximately a third of COPD patients, accounting for about 50% of deaths (Rabe et al., 2018), even after adjustment for confounding factors (Balbirsingh et al., 2022). The increased morbidity and mortality could be attributed to, at least in part, more frequent exacerbations (Balbirsingh et al., 2022; Rabe et al., 2018). The observation that both cardiovascular disease co‐morbidities and COPD have shared risk factors such as a history of cigarette smoking or exposure to noxious gas/particles, accelerated ageing and physical inactivity (Brassington et al., 2019), and that the degree of airflow obstruction may be an independent predictor of adverse cardiovascular outcomes (Sin et al., 2005) are suggestive of a causal relationship between airflow limitation and cardiovascular disease co‐morbidities in COPD (Sin et al., 2005). As both COPD and cardiovascular disease co‐morbidities represent a significant global impact on health, understanding the pathophysiology between them could potentially reduce this burden (Balbirsingh et al., 2022).

Although yet to be fully established, the ‘spill‐over’ of local lung inflammation in COPD has been demonstrated to drive cardiovascular disease risk (Barnes, 2019). In a preclinical model of COPD, Brassington et al. (2021) demonstrated that oxidative stress from cigarette smoke exposure may cause inflammatory cell infiltration into the vascular wall and endothelial dysfunction, suggesting oxidative stress may be a missing link. In addition to marking the imbalance between reactive oxygen species (ROS) and endogenous antioxidant defence, oxidative stress may simultaneously exacerbate co‐morbidities, stimulating fibrosis and emphysema development in the lungs by amplifying chronic inflammation (Barnes, 2020). The vascular endothelium is a single layer that lines the interior surface of the vessels, responsible for the regulation of vasomotor tone, vascular permeability, coagulation cascade, angiogenesis and innate and adaptive immunity (Brassington et al., 2019). Endothelial dysfunction is the earliest stage of cardiovascular disease co‐morbidities, characterised by an imbalance between endothelium‐derived vasodilating (i.e. nitric oxide [NO], prostacyclin [PGI_2_] and endothelium‐derived hyperpolarising factor [EDHF]) and vasoconstricting factors (i.e. endothelin‐1 and thromboxane) (Theodorakopoulou et al., 2021). In COPD, endothelial dysfunction may also be caused by the reduced bioavailability of ΝΟ from the inflammatory‐mediated changes of the vascular walls, such as degradation and replacement of the elastic fibres that controls vascular tone and function by collagen, increasing arterial stiffness and thus driving vascular dysfunction (Theodorakopoulou et al., 2021). Furthermore, persisting inflammation in COPD may also stimulate platelet activation, thereby establishing a pro‐coagulant environment in the vasculature (Hlapcic et al., 2020). Together, endothelial dysfunction and a pro‐coagulant environment promote arterial remodelling that may lead to pulmonary hypertension, a condition that worsens gas exchange and dyspnoea, as well as vulnerability to right ventricular dysfunction (Hlapcic et al., 2020). Therefore, addressing endothelial dysfunction is expected to reduce cardiovascular disease risk in COPD.

In the vasculature, NADPH oxidases (NOX) are considered the predominant sources of ROS generation that interferes with endothelial function (Brassington et al., 2019). Apocynin is a methoxy‐substituted catechol derived from the herb *Picrorhiza kurroa* exhibiting NOX inhibitory properties by blocking the assembly of a functional NOX complex and/or via radical scavenging action, depending on its concentration (Petronio et al., 2013). At a dosage of 5 mg·kg^−1^·day^−1^, we have previously demonstrated that apocynin exerted good anti‐inflammatory action and inhibitory effects on NOX2 (Oostwoud et al., 2016; Vlahos et al., 2011). Moreover, at this dosage, apocynin was able to prevent the loss of leg muscle mass and contractile function by cigarette smoke exposure (Chan et al., 2021), suggesting inhibition of oxidative stress and inflammation may lower cardiovascular disease risk in COPD. On this note, apocynin has been shown to restore endothelial function in streptozotocin‐induced diabetic rats (Olukman et al., 2010). However, its effectiveness against COPD‐related vascular impairment remains undetermined. In the present study, we aimed to define the effect of apocynin administration on the progression of COPD and vascular endothelial function following cigarette smoke exposure.

## 2 METHODS

### 2.1 Mice

Because of the strain dependence of the development of respiratory disease following cigarette smoke exposure (Vlahos et al., 2006), male Balb/C mice were chosen for this experiment. Male BALB/c mice (7 weeks of age) were obtained from the Animal Resources Centre (Perth, WA, Australia). Mice were housed in micro‐isolator cages (four mice per cage) at 21 ± 0.5°C on a 12‐h day/night cycle with *ad libitum* access to standard mouse chow diet and water. After 1 week of acclimatisation, mice were randomly assigned to one of the following equal size experimental groups:‐ room air (Sham) or cigarette smoke (CS) exposure groups, with or without daily supplementation of apocynin at 5 mg·kg^−1^ via intraperitoneal (i.p.) injection, n = 16 per group. Apocynin was prepared daily by dissolving in DMSO, before further dilution in sterile saline to reach a final concentration of ~0.05% DMSO. The vehicle groups were injected with saline (containing ~0.05% DMSO). Mice of the respective cages were weighed three times a week with daily monitoring. All experiments were conducted in accordance with the Australian Code of Practice for the Care of Experimental Animals and with RMIT University Animal Ethics Committee approval (Animal Ethics Application Number 1928). Animal studies are reported in compliance with the ARRIVE guidelines (Percie du Sert et al., 2020) and with the recommendations made by the *British Journal of Pharmacology* (Lilley et al., 2020).

### 2.2 Cigarette smoke exposure

Mice were placed in 18 L Perspex chambers and exposed to cigarette smoke from three cigarettes (Winfield Red, 16 mg or less of tar, 15 mg or less of carbon monoxide, 1.2 mg or less of nicotine; Philip Morris, Australia) spaced evenly over 1 h and carried out three times per day (09:00, 12:00 and 15:00 h), 5 days a week (Monday to Friday) for up to 24 weeks. The Sham mice were handled identically and exposed to room air. We have previously shown that this cigarette smoke exposure protocol in male BALB/c mice recapitulates key clinical traits of human COPD, including lung inflammation and pathology (airway inflammation, emphysema and impaired lung function), increased lung and systemic oxidative stress and co‐morbidities including skeletal muscle dysfunction (Chan et al., 2020; Dobric et al., 2022). Hence, only male mice were included in the present study.

### 2.3 Body composition scan and blood analysis

Fat and lean contents were determined using an EchoMRI apparatus (Echo Medical Systems, Houston, TX, USA). To understand the systemic impact of cigarette smoke exposure and apocynin treatment, whole blood was collected from the inferior vena cava at the end of the experiment and counted using a CELL‐DYN Emerald haematology analyser (Abbott Core Laboratory, USA), as previously described (Dobric et al., 2022). The mice were simply restrained for the duration of the scan (~5 min).

### 2.4 Lung function assessment, bronchoalveolar lavage and tissue collection

Mice were anesthetised by i.p. injection with ketamine (125 mg·kg^−1^) and xylazine (25 mg·kg^−1^) and tracheotomy was performed by inserting an 18G canular into the trachea. Lung function of anaesthetised mice was measured using a flexiVent ventilator (Scireq Inc. Montreal, Canada), which employs different scripts including force oscillation, deep inflation and constant phase models in mice with a breathing rate of 150 breaths per minute. Deflation of the lungs were performed to generate inspiratory capacity (IC). Pressure‐volume (PV) loops and negative pressure‐driven forced expiratory (NPFE) were performed to obtain inspiratory capacity (IC), static compliance, forced vital capacity (FVC), the pressure‐volume curve and the area enclosed by the pressure‐volume loop area as previously performed (Dobric et al., 2022). Following lung function testing, mice were killed by overdosing with sodium pentobarbitone (240 mg·kg^−1^) via i.p. injection. Body temperature was maintain by placing on a heated surface at 37°C.

Lungs were lavaged *in situ* using 0.4 ml of ice‐cold phosphate‐buffered saline (PBS) and three subsequent repeats of 0.3 ml PBS, with a return of approximately 1 ml of bronchoalveolar lavage fluid (BALF) per mouse as previously published (Chan et al., 2020, 2021). Bronchoalveolar lavage fluid (20 μl) was diluted 1:1 with Acridine Orange and the total number of viable cells counted on a standard Neubauer haemocytometer under fluorescent light on an Olympus BX53 microscope (Olympus Corporation, Tokyo, Japan). To differentiate cell populations in bronchoalveolar lavage fluid, cytocentrifuge preparations (Shandon Cytospin 3, 400 rpm, 10 min) were performed using ~5 × 10^4^ cells from the bronchoalveolar lavage fluid. Once dried, cells were fixed with Shandon™ Kwik‐Diff™ fixative (Thermofisher Scientific, NY, USA) and subsequently stained with Hemacolor® Rapid Red and Blue dye (Merck, Darmstadt, Germany) as per manufacturers' instructions, mounted with Entellan® New (Merck, Darmstadt, Germany). Cell types (macrophages, lymphocytes and neutrophils) were identified according to standard morphological criteria and at least 500 cells per slide were counted. After the lavage procedure, 10 ml of PBS was used to clear the lungs of blood via a right ventricular perfusion of the heart. Lung tissues were then collected, snap frozen in liquid nitrogen, and stored at −80°C until required.

### 2.5 Vascular myography

Vascular myography was used to assess vascular function as previously described (Brassington et al., 2021). Briefly, thoracic aortae were excised from the chest cavity, placed in a petri dish filled with carbogen‐bubbled (95% O_2_, 5% CO_2_) Krebs buffer (NaCl 119, KCl 4.7, MgSO_4_ 1.17, NaHCO_3_ 25, KH_2_PO_4_ 1.18, CaCl_2_ 2.5, glucose 5.5, all in mmol·L^−1^), before perivascular fat and connective tissue were carefully dissected away. The aortae were then cut into 2 mm rings and subject to vessel myography (*ex vivo* functional testing) using a 4 channel myograph unit (Model 610M, Danish Myo Technology (DMT) A/S, Denmark).

### 2.6 Histology and immunostaining

In a separate cohort of mice, the excised aortae were fixed in 10% Neutral Buffer Formalin (overnight at 4°C before subjecting to the Leica Tissue Processor (model ASP300, Leica Biosystems, MA, USA). The tissues were paraffin embedded using HistoCore Arcadia (Leica Biosystems, MA, USA), serial cross sections were cut using a microtome (Leica Biosystems, MA, USA) at 4 μm thickness onto Superfrost® Plus microscope slides (Thermofisher Scientific, NY, USA). The sections were dewaxed and rehydrated as previously described (Brassington et al., 2021, 2022), before antigen retrieval in sodium citrate buffer (10 mM Sodium Citrate, 0.05% Tween 20 at pH 6.0), blocking and permeabilization (PBS containing 1% bovine serum albumin, 22.52 mg·ml^−1^ glycine and 0.25% Triton X‐100) as described on https://www.abcam.com/protocols/immunocytochemistry-immunofluorescence-protocol.

For lung histology, haematoxylin and eosin (H&E), Alcian blue‐periodic acid Schiff (AB‐PAS) and Masson's trichrome stains were processed at Melbourne Histology Platform (University of Melbourne, Melbourne, VIC, Australia) and were performed for the assessment of tissue inflammation, airway mucus and airway collagen as previously described (Wang et al., 2021).

For aortic immunostaining, specific primary antibodies were used to detect vascular endothelial nitric oxide synthase (eNOS; anti‐NOS3 at 1:100 dilution; Thermo Fisher Scientific, USA), vascular peroxynitrite (anti‐3‐Nitrotyrosine [3‐NT] at 1:100 dilution; Thermofisher Scientific, NY, USA), platelet surface marker Integrin alpha 2b (anti‐CD41 at 1:100 dilution; Bioss Antibodies, MA, USA) and platelet activation marker p‐selectin (anti‐CD62p at 1:100 dilution; Bioss Antibodies, MA, USA). After an overnight incubation at 4°C, the sections were washed before exposure to fluorescent‐conjugated secondary antibody (Goat anti‐Rabbit IgG Fluor™ 488; Thermofisher Scientific, NY, USA) for 2 h at room temperature, light protected. After washing, the sections were coverslipped using Fluoromount‐G™ mounting medium containing DAPI (Thermofisher Scientific, NY, USA), imaged on an Olympus Slide Scanner (VS120‐S5, Olympus Corporation, Tokyo, Japan) and analysed using Olympus cellSens Dimension™ desktop software (version 1:18, Olympus Corporation, Tokyo, Japan). Positive signals (n = 8 per group) are expressed as fluorescence intensity fold change or area of positive stain as previously described (Brassington et al., 2021, 2022; Chan et al., 2020, 2021). All analysis was completed in a blinded manner. The Immuno‐related procedures used comply with the recommendations made by the *British Journal of Pharmacology* (Alexander et al., 2018).

### 2.7 Gene expression analysis

Total RNA was extracted from frozen lung tissue with a RNeasy Mini kit (Qiagen, MD, USA). cDNA was prepared with a High Capacity RNA‐to‐cDNA Kit (Thermofisher Scientific, NY, USA). qPCR was performed using pre‐developed TaqMan™ gene expression assays (Table 1; Thermofisher Scientific, NY, USA). The threshold cycle values (Ct) were normalised to a reference gene (glyceraldehyde phosphate dehydrogenase; GAPDH) and the relative fold change was determined by the ΔΔCt method (Chan et al., 2021; Vlahos et al., 2011).

**TABLE 1:** List of gene expression assays.

Gene name	Abbreviation	Taqman assay ID
Chemokine (C‐C motif) ligand 2	*Ccl2*	Mm00441242_m1
Chemokine (C‐X‐C motif) ligand 2	*Cxcl2*	Mm00436450_m1
Cytochrome b‐245, beta polypeptide (NADPH oxidase 2)	*Cybb*	Mm01287743_m1
Interleukin‐6	*Il6*	Mm00446190_m1
Tumour necrosis factor	*Tnfα*	Mm00443258_m1
Matrix metallopeptidase 12	*Mmp12*	Mm00500554_m1
NADPH oxidase 4	*Nox4*	Mm00479246_m1

### 2.8 Data and statistical analysis

The data and statistical analysis comply with the recommendations of the *British Journal of Pharmacology* on experimental design and analysis in pharmacology (Curtis et al., 2018). We have calculated mouse numbers to be eight mice per group for each output (myograph or immunostaining) using power calculations in DSS Researcher's Toolkit (α = 0.05; β = 0.2). Statistical analysis was done on n = 8 per group, where n is the number of indepedent values. Statistical analysis was undertaken only for studies where each group size was at least n = 5. Data sets were subjected to and passed normality test for Gaussian distribution using the D'Agostino & Pearson test, Anderson–Darling test, Shapiro–Wilk or Kolmogorov–Smirnov test. Data are presented as mean + standard errors of the mean (SEM) in the applicable absolute units or fold mean of the control (i.e. Sham Vehicle). Statistical differences between treatments were determined by two‐tailed unpaired Student’s *t* test or analysis of variance (ANOVA) followed by Tukey's multiple comparison post hoc tests where appropriate. One‐way ANOVA were used for three of more unmatched groups. Two‐way ANOVA were used to analyse data when response was influenced by two independent factors of interest. Post hoc tests were conducted only if F in ANOVA achieved *P*<0.05. All statistical analyses were performed using GraphPad Prism for Microsoft Windows® (Versions 9, GraphPad software®, CA, USA) where *P* < 0.05 was accepted as significant for all cases.

### 2.9 Materials

Apocynin (1‐(4‐Hydroxy‐3‐methoxyphenyl)ethan‐1‐one) and DMSO were obtained from Sigma‐Aldrich, MO, USA. The following drugs sodium pentobarbitone, ketamine and xylazine were obtained from Virbac (NSW, Australia), while the following chemicals sodium and potassium chloride, magnesium sulfate, sodium bicarbonate, sodium citrate, potassium hydrogen phosphate, calcium chloride and glucose along with acetylcholine, sodium nitroprusside and neutral buffer formalin were obtained from Sigma‐Aldrich (MO, USA). Details of other materials and suppliers were provided in the specific sections.

### 2.10 Nomenclature of targets and ligands

Key protein targets and ligands in this article are hyperlinked to corresponding entries in the IUPHAR/BPS Guide to PHARMACOLOGY http://www.guidetopharmacology.org and are permanently archived in the Concise Guide to PHARMACOLOGY 2021/22 (Alexander, Christopoulos, et al., 2021; Alexander, Fabbro, et al., 2021).

## 3 RESULTS

### 3.1 Apocynin treatment attenuated the deterioration in vascular endothelial function, vascular oxidative stress and injury by cigarette smoke exposure

Similar to our previous observations (Brassington et al., 2021; Chan et al., 2021), 8 weeks of cigarette smoke exposure resulted in marked airway inflammation evidenced by the 4.6‐fold increase in total number of cells present in the bronchoalveolar lavage fluid (Figure S1A), which are predominantly composed of macrophages and neutrophils, and lymphocytes (Figure S1B–D) to a minor extent. In line with the bronchoalveolar lavage fluid cell count, cigarette smoke exposure increased the expression of key pro‐inflammatory mediators:‐ *Tnfα*, *Il6* and *Mmp12* (Figure S1E–G) and chemo‐attractants:‐ *Ccl2* and *Cxcl2* (Figure S1H, I). Cigarette smoke exposure also increased the expression of *Cybb* (encodes for NOX2, Figure S1J) in the lungs, but not *Nox4* (Figure S1K), indicating a NOX2‐dependent nature of this cigarette smoke‐induced airway inflammation. Despite a 42% reduction in bronchoalveolar lavage fluid neutrophil count (Figure S1C), apocynin treatment had no significant effects on the expression of pro‐inflammatory mediators, chemo‐attractants or *Cybb* by cigarette smoke exposure (Figure S1E–K), suggesting the overall inflammatory status remained unchanged at this early time point.

**FIGURE 1:**
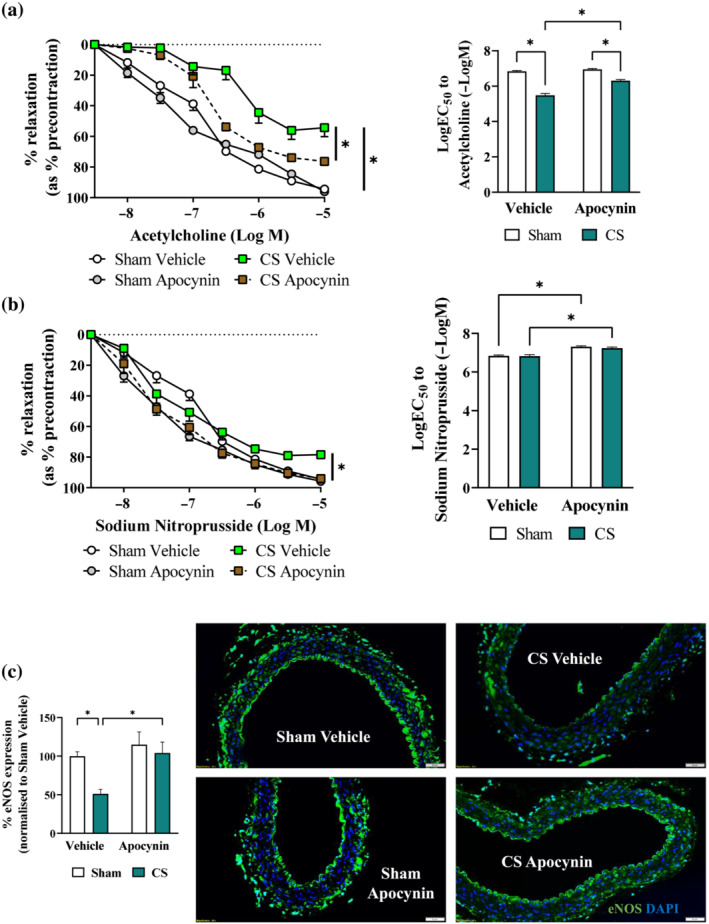
Cigarette smoke (CS)‐induced vascular dysfunction and platelet activation were attenuated by apocynin treatment. Mice were exposed to CV or room air (sham) for 8 weeks with i.p. injection of apocynin (5 mg·kg^−1^·day^−1^) or vehicle (saline). Cumulative concentration response curves and EC_50_ analysis to acetylcholine (a) or sodium nitroprusside (b) (1 × 10^−8^ M to 1 × 10^−5^ M) to examine endothelial‐dependent and endothelial‐independent vasodilation in mouse thoracic aorta, respectively. Representative immunofluorescence images of thoracic aorta section stained with endothelial nitric oxide synthase (eNOS; c) data are expressed as mean + SEM (n = 8 mice per group) and analysed by two‐way ANOVA with multiple comparisons and Tukey post hoc test. *P < 0.05, denotes differences between the compared groups.

**FIGURE 2:**
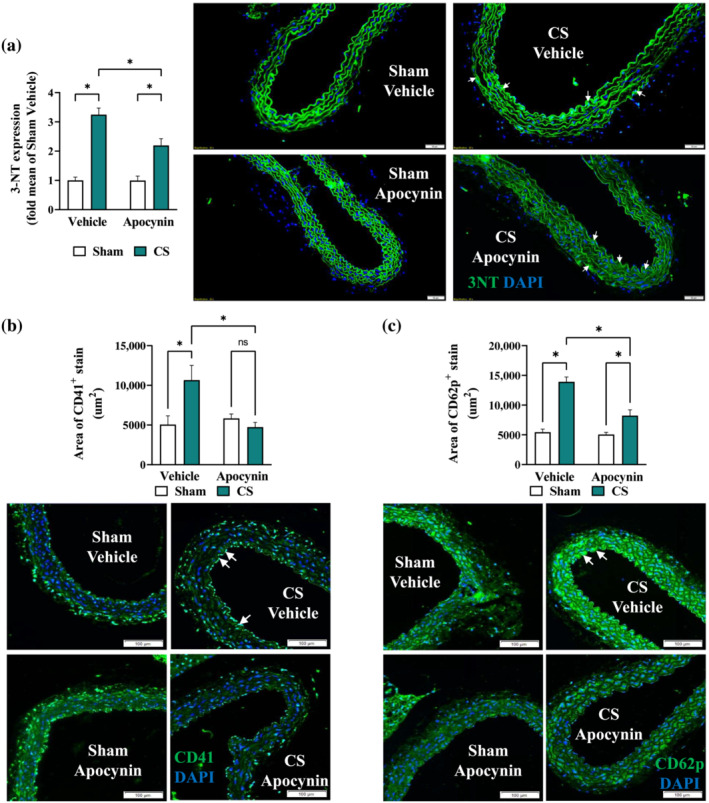
Cigarette smoke (CS)‐induced vascular oxidative stress and platelet aggregation were prevented by apocynin treatment. Mice were exposed to CS or room air (sham) for 8 weeks with i.p. injection of apocynin (5 mg·kg^−1^·day^−1^) or vehicle (saline). Representative immunofluorescence images of thoracic aorta section stained with 3‐nitrotyrosine (3‐NT; a), platelet surface marker (CD41; b), or platelet activation marker (CD62p; c), with positive staining in green and nuclei in blue (DAPI). White arrows indicate positive stains and the respective positive stains were quantified as described in methods. Data are expressed as mean + SEM (n = 8 mice per group) and analysed by two‐way ANOVA with multiple comparisons and Tukey post hoc test. *P < 0.05 denotes differences between the compared groups.

To assess the effects of apocynin treatment on vascular function, precontracted thoracic aorta segments were exposed to increasing concentrations of acetylcholine to examine endothelial‐dependent relaxation responses. Cigarette smoke exposure significantly impaired aortic vasodilation to acetylcholine, evidenced by the altered acetylcholine relaxation curve and an increased EC_50_ (Figure 1a) when compared with Sham Vehicle. Meanwhile, smooth muscle‐dependent aortic relaxation induced by sodium nitroprusside (SNP), a NO donor, was seemingly unaffected by cigarette smoke exposure, albeit a 20% reduction in maximal response (i.e. R_max_ at 10^−5^ M) was detected (Figure 1b). These findings suggest impairment of endothelial‐dependent vasodilation by cigarette smoke exposure. In line with this, our immunostaining detected a 50% reduction in vascular eNOS expression (Figure 1c), together with a concomitant three‐fold increase in 3‐nitrotyrosine (3‐NT; Figure 2a) by cigarette smoke exposure, suggesting a reduced bioavailability of endothelial NO and the presence of vascular oxidative stress. In line with this, increased adhesion of platelets to the endothelium and their activation were observed, marked by the increased fluorescence signal of CD41 (Figure 2b) and CD62p (Figure 2c), respectively, suggesting vascular injury. Apocynin treatment improved both the acetylcholine‐stimulated and SNP‐induced vasodilatory response (Figure 1a,b), which were associated with preserved eNOS expression (Figure 1c) and lessened vascular oxidative stress (Figure 2a). Thus reducing the adhesion and activation of platelets at the vascular endothelium (Figure 2b,c). Meanwhile, no significant effects were found on the heart weight following cigarette smoke exposure and/or apocynin treatment (Figure S1L), suggesting the cardiovascular defects in our model are mainly located in the vasculature, which is the focus for the rest of the study.

### 3.2 Apocynin treatment attenuated the suppressive effect of cigarette smoke exposure on body weight gain and lymphocytosis

**FIGURE 3:**
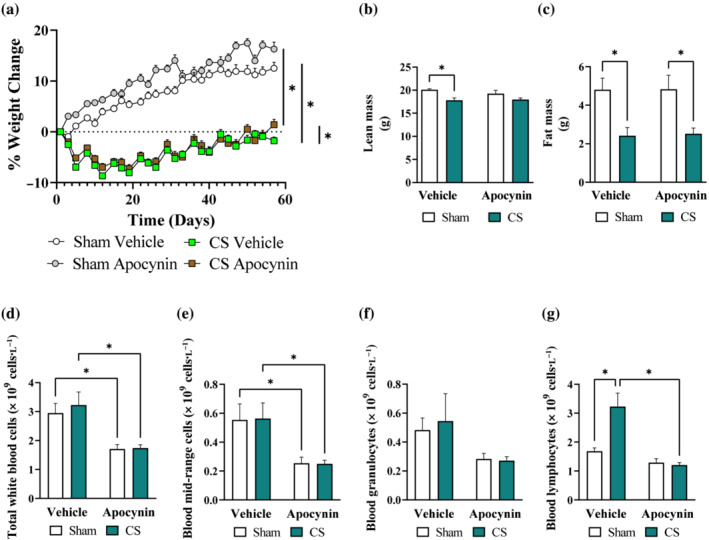
Cigarette smoke (CS)‐induced loss of lean mass and systemic lymphocytosis were prevented by apocynin treatment. Mice were exposed to CS or room air (sham) for 8 weeks with i.p. injection of apocynin (5 mg·kg^−1^·day^−1^) or vehicle (saline). Change in body weight (a), alterations in body lean (b) and fat (c) mass. Haematology analysis of total white blood cells (d), mid‐range cells (e), granulocytes (f) and lymphocytes (g). Data are expressed as mean + SEM (n = 8 mice per group) and analysed by two‐way ANOVA with multiple comparisons and Tukey post hoc test. *P < 0.05, denotes differences between the compared groups.

Consistent with the suppressive effects on weight gain observed in humans (Chan et al., 2019), chronic cigarette smoke exposure markedly reduced body weight gain in mice (Figure 3a), which was attributed to ~12% loss in overall lean mass (Figure 3b) and ~50% loss in overall fat mass (Figure 3c). Despite the lack of effects on fat mass, apocynin treatment attenuated the suppressive effect of cigarette smoke exposure on lean mass (Figure 3b,c). This suggested that apocynin treatment assisted in the preservation of lean mass against cigarette smoke exposure, consistent with the improved body weight observed in the apocynin‐treated animals at the end of the study (Figure 3a). In contrast to the bronchoalveolar lavage fluid data, cigarette smoke exposure specifically increased (two‐fold) blood lymphocyte counts (Figure 3d–g), marking the systemic lymphocytosis and chronic inflammation. Consistent with its renowned antioxidant and anti‐inflammatory effects, apocynin treatment attenuated cigarette smoke‐induced lymphocytosis.

### 3.3 Long term apocynin treatment reduced lung function decline, airway fibrosis and inflammation caused by cigarette smoke exposure

**FIGURE 4:**
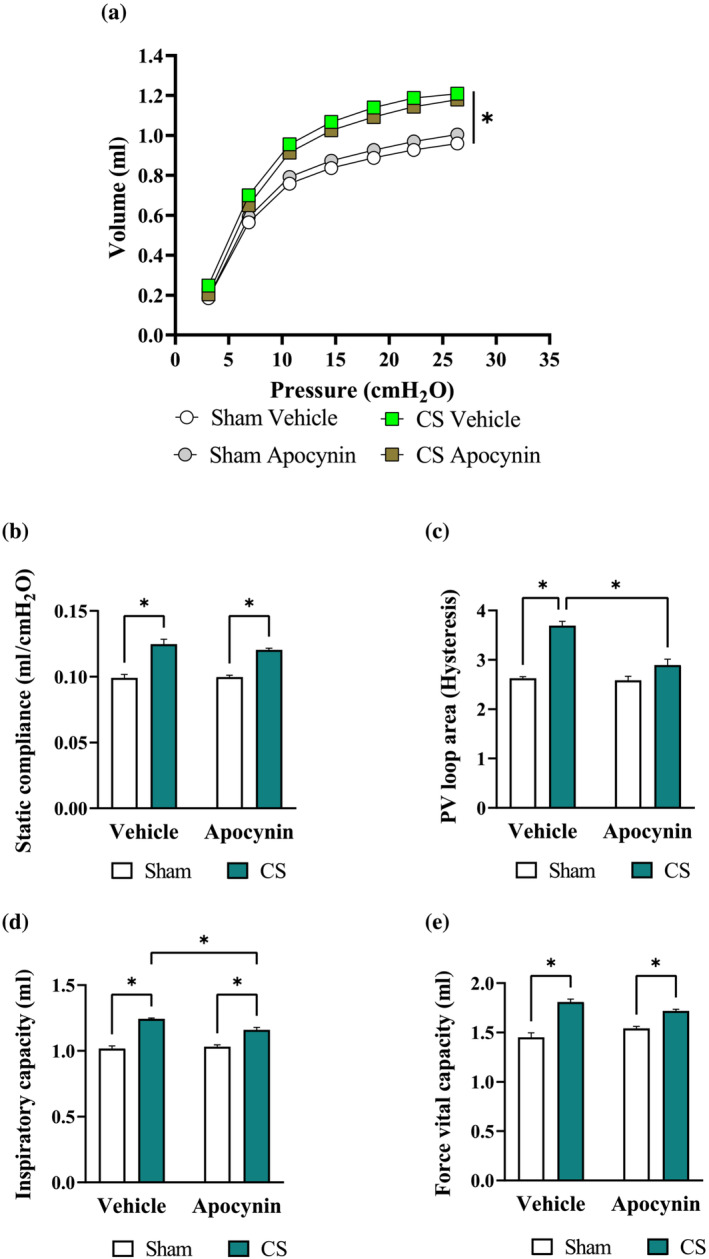
The cigarette smoke (CS)‐induced lung hyperinflation was lessened by apocynin treatment. Mice were exposed to CS or room air (sham) for 8 weeks with i.p. injection of apocynin (5 mg·kg^−1^·day^−1^) or vehicle (saline). Different respiratory parameters from the lung function assessment, including pressure‐volume (PV) ratio (a), static compliance (b), PV loop area (c), inspiratory capacity (IC; d), and forced vital capacity (FVC; e). Data are expressed as mean + SEM (n = 8 mice per group) and analysed by two‐way ANOVA with multiple comparisons and Tukey post hoc test. *P < 0.05 denotes differences between the compared groups.

**FIGURE 5:**
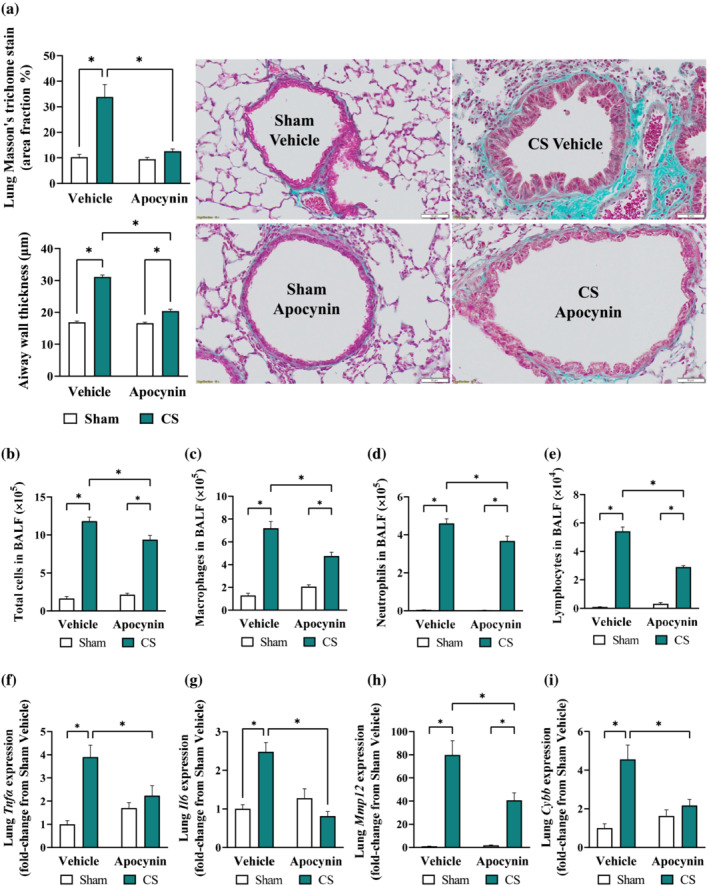
Long term cigarette smoke (CS)‐induced airway inflammation and fibrosis from were attenuated by apocynin treatment. Mice were exposed to CS or room air (sham) for 8 weeks with i.p. injection of apocynin (5 mg·kg^−1^·day^−1^) or vehicle (saline). Representative Masson's trichome staining of cross‐lung sections for collagen deposition surrounding smaller airways and airway wall thickening (a). Total number of cells (b), macrophage (c), neutrophils (d), and lymphocytes (e) in the bronchoalveolar lavage fluid (BALF). Quantitative PCR was performed to assess the expression of *Tnfα* (f), *Il6* (g), *Mmp12* (h) and *Cybb* (i) in homogenised lung tissues. Data are expressed as mean + SEM (n = 8 mice per group) and analysed by two‐way ANOVA with multiple comparisons and Tukey post hoc test. *P < 0.05 denotes differences between the compared groups.

The promising benefits observed in the 8‐week protocol have prompted us to investigate whether apocynin administration may alter the development of COPD. Cigarette smoke exposure for 24 weeks resulted in lung function decline and emphysema marked by a left‐shift in the pressure‐volume (PV)‐loop curve (Figure 4a) as well as increases in static compliance (Figure 4b), pressure‐volume loop area (Figure 4c), inspiratory capacity (IC; Figure 4d) and forced vital capacity (FVC; Figure 4e), suggesting hyperinflation of the lungs. The increase of pressure‐volume loop area and inspiratory capacity were significantly reduced by apocynin treatment. Apocynin treatment also inhibited the thickening of the small airway epithelium and excessive collagen deposition caused by cigarette smoke exposure (Figure 5a), which is associated with airway stiffening (Liu et al., 2021). Despite the renowned effect of habitual smoking on airway mucus hypersecretion in humans (Allinson et al., 2016), no mucus congestions were found in the airways of cigarette smoke‐exposed mice (Figure S2), suggesting mucus hypersecretion is unlikely to be driven by cigarette smoke exposure per se. Compared to the 8‐week protocol, apocynin treatment had a more noticeable benefit on cigarette smoke‐induced airway inflammation, evidenced by a reduction in total number of cells (by 21%), macrophages (by 33%), neutrophil (by 20%) and lymphocytes (by 47%) counts in the bronchoalveolar lavage fluid (Figure 5b–e). Accordingly, the cigarette smoke‐induced expression of pro‐inflammatory mediators (*Tnfα*, *Il6* and *Mmp12*; Figure 5f–h) and *Cybb* (Figure 5i) were significantly reduced by apocynin treatment.

### 3.4 Long term apocynin treatment preserved vascular endothelial function against cigarette smoke exposure

**FIGURE 6:**
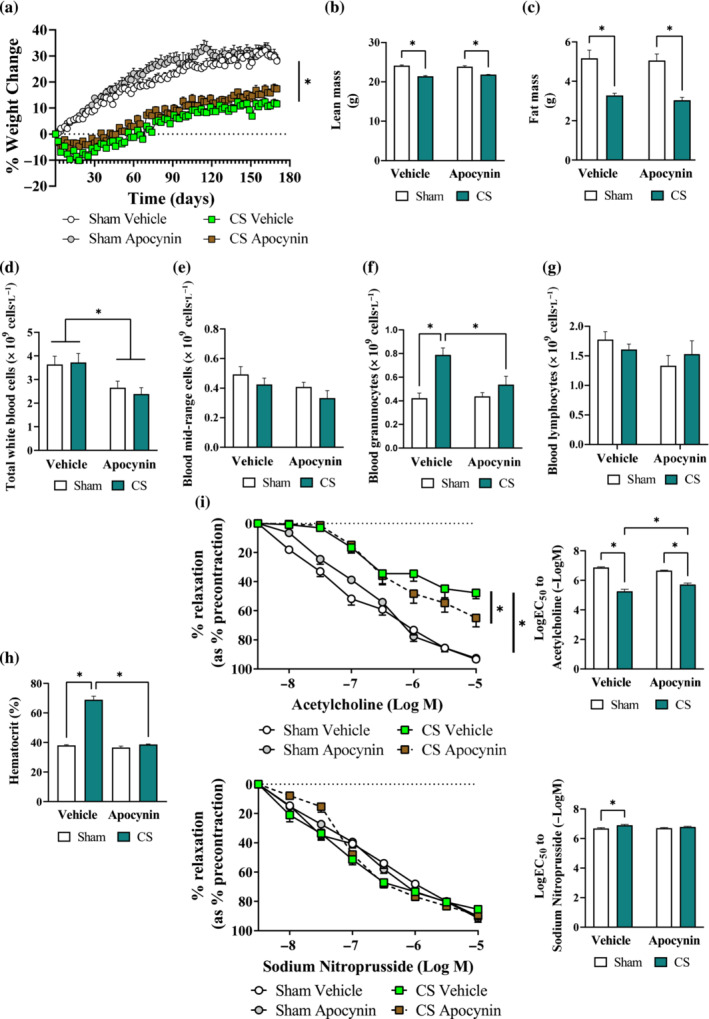
Long term cigarette smoke (CS)‐induced systemic neutrophilia and vascular dysfunction were attenuated by apocynin treatment. Mice were exposed to CS or room air (sham) for 8 weeks with i.p. injection of apocynin (5 mg·kg^−1^·day^−1^) or vehicle (saline). Change in body weight (a), alterations in body lean (b) and fat (c) mass. Haematology analysis of total white blood cell (d), monocytes (e), neutrophils (f), lymphocytes (g) and haematocrit (h). Cumulative concentration response curves and EC_50_ analysis to acetylcholine and sodium nitroprusside (i; 1 × 10^−8^M to 1 × 10^−5^M) to examine endothelial‐dependent and endothelial‐independent vasodilation in mouse thoracic aorta, respectively. Data are expressed as mean + SEM (n = 8 mice per group) and analysed by two‐way ANOVA with multiple comparisons and Tukey post hoc test. *P < 0.05, denotes differences between the compared groups.

**FIGURE 7:**
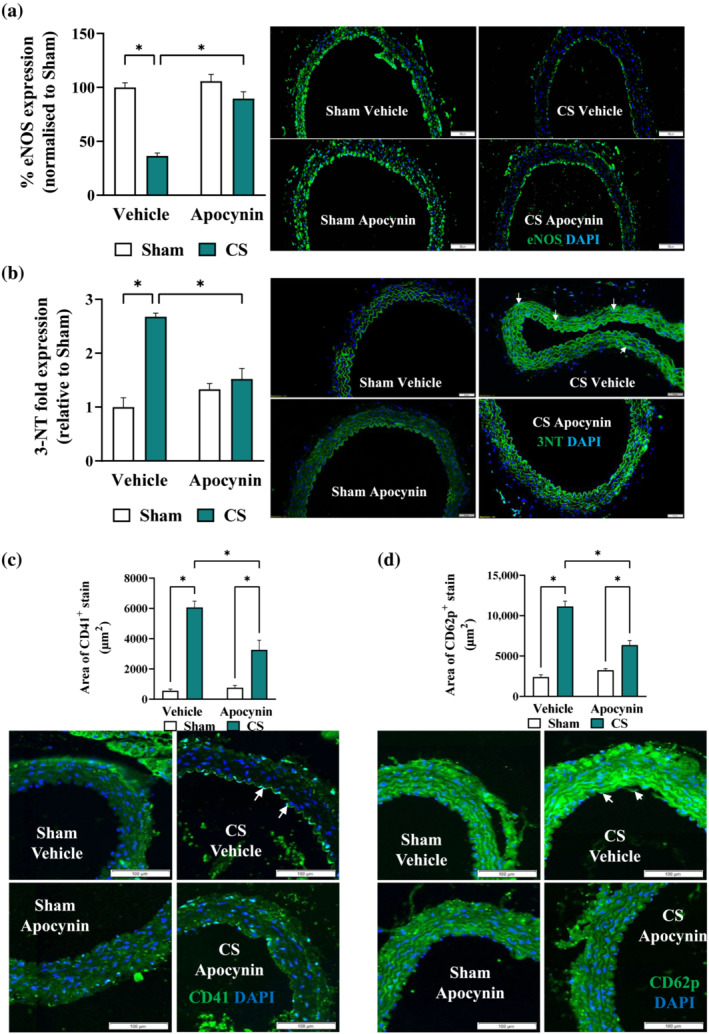
Long term cigarette smoke (CS)‐induced loss of eNOS, vascular oxidative stress and platelet activation were attenuated by apocynin treatment. Mice were exposed to CS or room air (sham) for 8 weeks with i.p. injection of apocynin (5 mg·kg^−1^·day^−1^) or vehicle (saline). Representative immunofluorescence images of thoracic aorta section stained with eNOS (a), 3‐NT (b), platelet surface CD41 (c), or platelet activation CD62p (d), with positive staining in green and nuclei in blue (DAPI). White arrows indicate positive stains and the respective positive stains were quantified as described in methods. Data are expressed as mean + SEM (n = 8 mice per group) and analysed by two‐way ANOVA with multiple comparisons and Tukey post hoc test. *P < 0.05, denotes differences between the compared groups.

Similar to that of the 8‐week protocol, mice exposed to cigarette smoke for 24 weeks displayed ~67% reduction in weight gain compared to the Sham group (Figure 6a), attributing to ~13% and ~36% loss in lean and fat mass, respectively (Figure 6b,c), which were unaltered by apocynin treatment. Unlike that of the 8‐week protocol, 24 weeks of cigarette smoke exposure resulted in a two‐fold increase in blood granulocytes count which was prevented by apocynin treatment (Figure 6d–g). Apocynin treatment also prevented the cigarette smoke‐induced increase in haematocrit (Figure 6h), which is an indicator of chronic hypoxia (Kent et al., 2011). Similar to that of the 8‐week protocol, cigarette smoke exposure caused a marked impairment in the acetylcholine‐mediated vasodilation, leaving the SNP‐stimulated vasodilation unaltered (Figure 6i). In agreement with this, eNOS expression (Figure 7a) was diminished by 24 weeks of cigarette smoke exposure, which may be attributed to an increased oxidative modifications of the aorta (Figure 7b). This was accompanied by increased platelet adhesion (CD41) and activation (CD62p) at the vascular endothelium (Figure 7c,d). Apocynin treatment preserved maximal acetylcholine‐mediated vasodilation by ~60% (maximal relaxation response [R_max_]; Figure 6i) in cigarette smoke‐exposed mice. In line with this, the availability of eNOS, oxidative modifications of the aorta and platelet adhesion/activation (Figure 7a–d) were normalised, suggesting the inhibitory effects of apocynin treatment may offer long‐term protection against vascular oxidative stress and endothelial dysfunction.

## 4 DISCUSSION

The present study sought to understand the benefits of apocynin treatment on cigarette smoke‐induced pulmonary and vascular pathologies. Cigarette smoke exposure caused significant airway inflammation, vascular dysfunction and platelet reactivity. By limiting oxidative stress, apocynin treatment attenuated airway inflammation. and prevented the onset of both vascular dysfunction and platelet reactivity by cigarette smoke exposure. We found that long‐term apocynin treatment had marked benefits on airway inflammation and attenuated the progression of COPD, with reduced pulmonary hyperinflation and airway fibrosis arising from chronic cigarette smoke exposure. These findings support modulating oxidative stress as viable strategies for the management of COPD.

Chronic inflammation is the most important feature of cigarette smoke exposure and the strongest driver of pulmonary (Hikichi et al., 2019) and systemic pathologies (Brassington et al., 2019; Chan et al., 2019) in COPD. The chronic inflammatory process in COPD involves both innate and adaptive immunity and is most pronounced in the bronchial walls of the small airways (King, 2015). Chronic inflammation of the airway leads to the accumulation of inflammatory mucus exudates in the lumen and increased tissue volume of the bronchial wall (Cosio et al., 2009). The increased tissue volume of the bronchial wall is a result of infiltration by both innate (macrophages and neutrophils) and adaptive immune cells (lymphocytes), leading to the airflow obstruction/chronic bronchitis seen in COPD (Barnes, 2016). Meanwhile the development of emphysema appears to be closely linked to the infiltration of macrophages and neutrophils into the lung parenchyma (Barnes, 2020). Cigarette smoke exposure increases their recruitment and activation in the respiratory tract, leading to the production of various proinflammatory mediators, including cytokines/chemokines (TNF‐α, IL‐6 and CCL2), ROS (superoxide and NO), and proteases (MMP9 and 12) (Stampfli & Anderson, 2009) that drives the development of emphysema (Barnes, 2016). The production of these proinflammatory mediators is triggered by the activation of toll like receptors (TLRs) and lymphocyte antigen receptors, resulting in the activation of the signal transducers and activators of transcription (STATs)/nuclear factor kappa B (NF‐κβ) intracellular signalling pathways (Vlahos & Bozinovski, 2015, 2018). In line with this, our preclinical model recapitulated the chronic airway inflammation, immune cell infiltration and the consequential decline in lung function and emphysema manifestation at the 6‐month time point. Strikingly, cigarette smoke exposure was associated with a distinct increase in blood lymphocyte count (at 2‐month) and neutrophil count (at 6‐month), suggesting the driving force of systemic inflammation in COPD is unlikely to be mutually exclusive and may involve the dynamic action of inflammatory cells from both innate and adaptive immunity. On this note, it is understood that the inflammatory process in COPD has marked heterogeneity (King, 2015) depending on several extrinsic factors such as smoke history, age, lifestyle and current medications (Alabi et al., 2021). This is further complicated by acute exacerbations which may lead to several different endotypes driven by neutrophils, eosinophils, dendritic cells and mast cells (Barnes, 2019). Our results highlight that chronic cigarette smoke exposure per se may produce distinct systemic inflammatory profile independent of exacerbations or the influence of the aforementioned extrinsic factors.

It is well‐established that COPD increases the risk of cardiovascular disease by two‐ to four‐fold (Shaw et al., 2014), which may be attributed to systemic inflammation (Thomsen et al., 2012). Clinically, systemic inflammation in COPD is often identified by the increase of several biomarkers in the circulation, such as C‐reactive protein (CRP), IL‐6, IL‐8 (CXCL8), TNF‐α, fibrinogen and leukocyte (Cardoso et al., 2021). However, it is noteworthy that systemic inflammation is not a stable feature in all patients with COPD, with considerable variations from time to time even within the same individual (Cardoso et al., 2021). In 8656 patients with COPD from two large Danish population, Thomsen et al (2012) observed a greater risk of cardiovascular disease‐related hospitalisation or death compared with the non‐COPD counterparts, despite only 10% of the COPD patients displaying elevated levels of all three of the biomarkers measured (CRP, fibrinogen and leukocyte), suggesting the predictive power of these biomarkers on cardiovascular disease risk in COPD, even when presented *in silos*. In line with the increased cardiovascular disease risk, mice exposed to cigarette smoke displayed impaired vasodilation. Impaired vasodilation can be a result of either the endothelium failing to send vasodilatory signals to the smooth muscle due to the loss of endothelial layer integrity (endothelial‐dependent) or the smooth muscle cells not responding to endothelial signalling and dilate (endothelial‐independent). Our result suggested that cigarette smoke exposure specifically dampens eNOS action and endothelium‐dependent vasodilation, while the vascular smooth muscle dilatory response was unaffected, regardless of cigarette smoke exposure duration. This is somewhat surprising, as nicotine has been shown to diminish endothelium‐independent vasodilation via the production of superoxide anion, which impairs the action of ATP‐sensitive K^+^ channels on voltage gated Ca^2+^ channels (Mayhan & Sharpe, 2002). Moreover, cigarette smoke‐derived nicotine has been shown to enhance vasoconstrictive response of the vascular smooth muscle to phenylephrine (Olfert et al., 2018; Sarabi & Lind, 2000), suggesting its amplifying effect on α_1_‐adrenoceptor activation. However, studies in mice (Brassington et al., 2021; Olfert et al., 2018) and smokers (Sarabi & Lind, 2000) have found that SNP‐induced vasodilation is generally preserved. The reason for this discrepancy is unclear, though recent work by Oakes et al (Oakes et al., 2020) demonstrated that chronic nicotine exposure also increases the body's tolerance to the hypertensive effects of nicotine, which may explain the seemingly unaltered SNP‐induced vasodilation in our study.

In addition to vasodilation impairment, increased platelet reactivity may also arise form habitual smoking (Brassington et al., 2019). Increased platelet reactivity promotes dysregulated platelet aggregation which is associated with increased risk of myocardial infarction, terminal occlusive thrombotic events and sudden death in patients with COPD (Brassington et al., 2019). Firstly, cigarette smoke exposure has been shown to directly cause platelet activation and their aggregation via the activation of the cyclooxygenase‐1 pathway (Loke et al., 2014). Secondly, the spill‐over of pro‐inflammatory mediators from the COPD lungs into the vasculature may causes the adhesion of activated platelets to the arterial wall and collagen fibres, via the up‐regulation of CD41, CD62p (p‐selectin) and von Willebrand factor (Jennings, 2009). Thirdly, endothelial dysfunction constitutes vascular injury which may stimulate platelets aggregation and adhesion to the endothelium, releasing platelet‐derived growth factors to promote formation of thrombo‐embolism consisting of platelets, endothelial cells, monocytes and erythrocytes at the site of the injury (Hamilos et al., 2018). Of interest, both platelet aggregation (Pamukcu et al., 2011) and platelet‐derived cyclooxygenase‐1 (COX‐1) expression (Loke et al., 2014) were markedly elevated in habitual smokers, independent of acute smoking. This suggest platelet activation and aggregation is likely to be a consequence of long‐term repeated exposure to cigarette smoke.

In line with its antioxidant and anti‐inflammatory properties (Petronio et al., 2013), both cigarette smoke‐induced airway and systemic inflammation were effectively reduced by apocynin treatment, particularly with long‐term administration. Although lung function decline and emphysema remained evidenced, apocynin treatment lessened hyperinflation of the lungs, which was associated with a marked reduction in epithelium thickening and fibrosis of the small airway. Hyperinflation has been shown to reduce gas exchange capacity, which results in persisting hypoxia, eliciting adverse pulmonary vascular remodelling and pulmonary hypertension (Dunham‐Snary et al., 2017). In line with this, we detected an increase in haematocrit percentage, suggesting the presence of hypoxemia following long‐term cigarette smoke exposure. Meanwhile, hyperinflation is caused by air trapping which is strongly linked to increase intensity of dyspnoea (Barnes, 2019). Together, both hypoxemia and dyspnoea form a vicious spiral of activity avoidance, physical deconditioning and reduced quality of life, thereby accelerating the development of extra‐pulmonary co‐morbidities such as cardiovascular disease (Rabe et al., 2018). Clinically, long‐acting bronchodilator treatment has been shown to reduce hyperinflation in COPD (Di Marco et al., 2018). Our study demonstrated that apocynin treatment reduced both of these cigarette smoke‐induced impairments, suggesting apocynin may be used in conjunction with long‐acting bronchodilator to improve gas‐exchange and reduce exertional dyspnoea in COPD. This in turn would increase a patient's ability to exercise and their likelihood to benefit from pulmonary rehabilitation. In terms of vascular function, apocynin treatment offered complete preservation of eNOS expression and endothelium‐dependent vasodilation against continuous cigarette smoke exposure. This finding supports the notion that limiting oxidative stress and systemic inflammation as a viable treatment strategy for pulmonary and systemic co‐morbidities in COPD. More importantly, our study also raises the concept that oxidative stress should be incorporated as a part of the new treatable trait approach for COPD (Cardoso et al., 2021; Duszyk et al., 2021), given oxidative stress and inflammation are known to perpetuate each other (Barnes, 2022). Therefore, treatments that disrupt/stop this vicious cycle should benefit people with COPD, much similar to that of apocynin treatment in our preclinical COPD model.

By limiting oxidative stress and inflammation, our data suggested that apocynin lowers the influx of immune‐cells and cytokine release from repeated cigarette smoke exposure and prevented endothelial dysfunction in the thoracic aorta, which has several implications on the pathogenesis of COPD. Firstly, the lessened recruitment of neutrophils and lymphocytes, and cytokine release alleviated lung hyperinflation, thereby altering the course of lung function decline. Secondly, the extensive vascular networks embedded within the alveoli enables the rapid spread of inflammation and oxidative stress to the vasculature (Balbirsingh et al., 2022), particularly the thoracic aortae, leading to endothelial dysfunction. The attenuated vascular dysfunction and pathology imply that apocynin was effective in limiting the spread of inflammation and oxidative stress, thus protecting against the onset of cardiovascular co‐morbidities in COPD. Thirdly, the extensive vascular networks within the alveoli also imply cellular crosstalk between vascular endothelium and pneumocytes, which is an integral component of the epithelial‐mesenchymal trophic unit (EMTU). In addition to endothelial dysfunction, repeated vascular injury provokes endothelial cells to acquire mesenchymal phenotype, expressing various markers (e.g. alpha‐smooth muscle actin, vimentin and fibronectin) that would drive aberrant activation of the epithelial‐mesenchymal trophic unit (Osei & Hackett, 2020). In particular, the aberrant activation of type II pneumocytes produce extracellular matrix proteins including collagen, fibronectin and MMPs for airway remodelling and fibrosis (Osei & Hackett, 2020). Our molecular and morphological data confirmed the presence of airway remodelling and fibrosis by chronic cigarette smoke exposure. Meanwhile, the preventative effects of apocynin on both vascular and airway pathologies support the aberrant crosstalk between the vasculature and epithelial‐mesenchymal trophic unit as a driving force, thus providing another rationale for oxidative stress modulating therapy in the management of COPD.

In summary, the present study demonstrated that habitual cigarette smoke exposure is linked to vascular dysfunction and increased platelet reactivity *in vivo*, which may be alleviated by the inhibition of oxidative stress. Both airway and systemic inflammation are recognised treatable traits of COPD, however oxidative stress is surprisingly yet to be included. Clinically, oxidative stress may be measured by a number of biomarkers present in exhaled breath such as 8‐isoprostane (Montuschi et al., 2000), H_2_O_2_ (Nowak et al., 2001), nitric oxide metabolites (NO^2−^/NO^3−^) (Balint et al., 2001), and/or those from blood like malondialdehyde (Lykkesfeldt et al., 2004), thiobarbituric acid reactive substances (Del Rio et al., 2005), carbonyl derivatives (Zinellu et al., 2016), and ferric ion levels (Zinellu et al., 2016). Future studies should examine the clinical feasibility and benefit of treating oxidative stress on key health outcomes, including 6‐min walk distance, spirometry and health‐related quality of life score.

## AUTHOR CONTRIBUTIONS

**Stanley M. H. Chan:** Conceptualization (supporting); data curation (equal); formal analysis (equal); investigation (equal); methodology (equal); supervision (equal); validation (supporting); writing—original draft (lead); writing—review and editing (equal). **Kurt Brassington:** Data curation (equal); formal analysis (equal); investigation (equal); methodology (supporting); writing—review and editing (supporting). **Suleman Abdullah Almerdasi:** Data curation (supporting); methodology (supporting); writing—review and editing (supporting). **Aleksandar Dobric:** Data curation (supporting); formal analysis (supporting); investigation (equal); methodology (equal); writing—review and editing (supporting). **Simone N. De Luca:** Conceptualization (equal); data curation (supporting); formal analysis (supporting); investigation (equal); methodology (supporting); supervision (equal); validation (supporting); writing—original draft (supporting); writing—review and editing (supporting). **Madison Coward‐Smith:** Data curation (supporting); investigation (supporting); methodology (supporting). **Hao Wang:** Conceptualization (supporting); data curation (supporting); formal analysis (supporting); investigation (supporting); methodology (supporting); writing—review and editing (supporting). **Kevin Mou:** Data curation (supporting); investigation (supporting); methodology (supporting); writing—review and editing (supporting). **Alina Akhtar:** Data curation (supporting); methodology (supporting); writing—review and editing (supporting). **Rana Abdullah Alateeq:** Data curation (supporting); methodology (supporting); writing—review and editing (supporting). **Wei Wang:** Data curation (supporting); investigation (supporting); methodology (supporting); writing—review and editing (supporting). **Huei Jiunn Seow:** Conceptualization (supporting); data curation (supporting); formal analysis (supporting); investigation (supporting); methodology (supporting); project administration (supporting); validation (supporting); writing—review and editing (supporting). **Stavros Selemidis:** Data curation (supporting); investigation (supporting); methodology (supporting); writing—review and editing (supporting). **Steven Bozinovski:** Data curation (supporting); funding acquisition (supporting); investigation (supporting); methodology; validation (supporting); writing—review and editing (supporting). **Ross Vlahos:** Conceptualization (lead); data curation (equal); formal analysis (equal); funding acquisition (lead); investigation (equal); methodology (equal); project administration (lead); resources (lead); software (lead); supervision (lead); validation (lead); visualization (lead); writing—original draft (equal); writing—review and editing (equal).

## CONFLICT OF INTEREST STATEMENT

The authors declare no conflict of interest in this study.

## DECLARATION OF TRANSPARENCY AND SCIENTIFIC RIGOUR

This Declaration acknowledges that this paper adheres to the principles for transparent reporting and scientific rigour of preclinical research as stated in the *BJP* guidelines for Design & Analysis, Immunoblotting and Immunochemistry and Animal Experimentation and as recommended by funding agencies, publishers and other organizations engaged with supporting research.

## Supporting information


**Figure S1.** Bronchoalveolar lavage fluid (BALF) cellularity (A‐D), markers of oxidative stress and inflammation (E‐K) and heart weight (L) of 8‐week cigarette smoke (CS) exposed mice with or without daily apocynin administration.
**Figure S2.** Representative images of lung sections stained with Alcian blue‐periodic acid Schiff for mucus secretion. Kidney section was included as positive control.

## Data Availability

The data that support the findings of this study are available from the corresponding author upon reasonable request. Some data may not be made available because of privacy or ethical restrictions.
