# Supplementary material for: Inhibition of oxidative stress by apocynin attenuated chronic obstructive pulmonary disease progression and vascular injury by cigarette smoke exposure
Source: Br J Pharmacol. 2023 Mar 31;180(15):2018–34. doi: 10.1111/bph.16068 (PMC10953324; doi:10.1111/bph.16068)
Supplement: Supplementary file 1 — Figure S1. Bronchoalveolar lavage fluid (BALF) cellularity (A‐D), markers of oxidative stress and inflammation (E‐K) and heart weight (L) of 8‐week cigarette smoke (CS) exposed mice with or without daily apocynin administration. Figure S2. Representative images of lung sections stained with Alcian blue‐periodic acid Schiff for mucus secretion. Kidney section was included as positive control. [file BPH-180-2018-s001.pdf]

Figure S1: 8-week CS

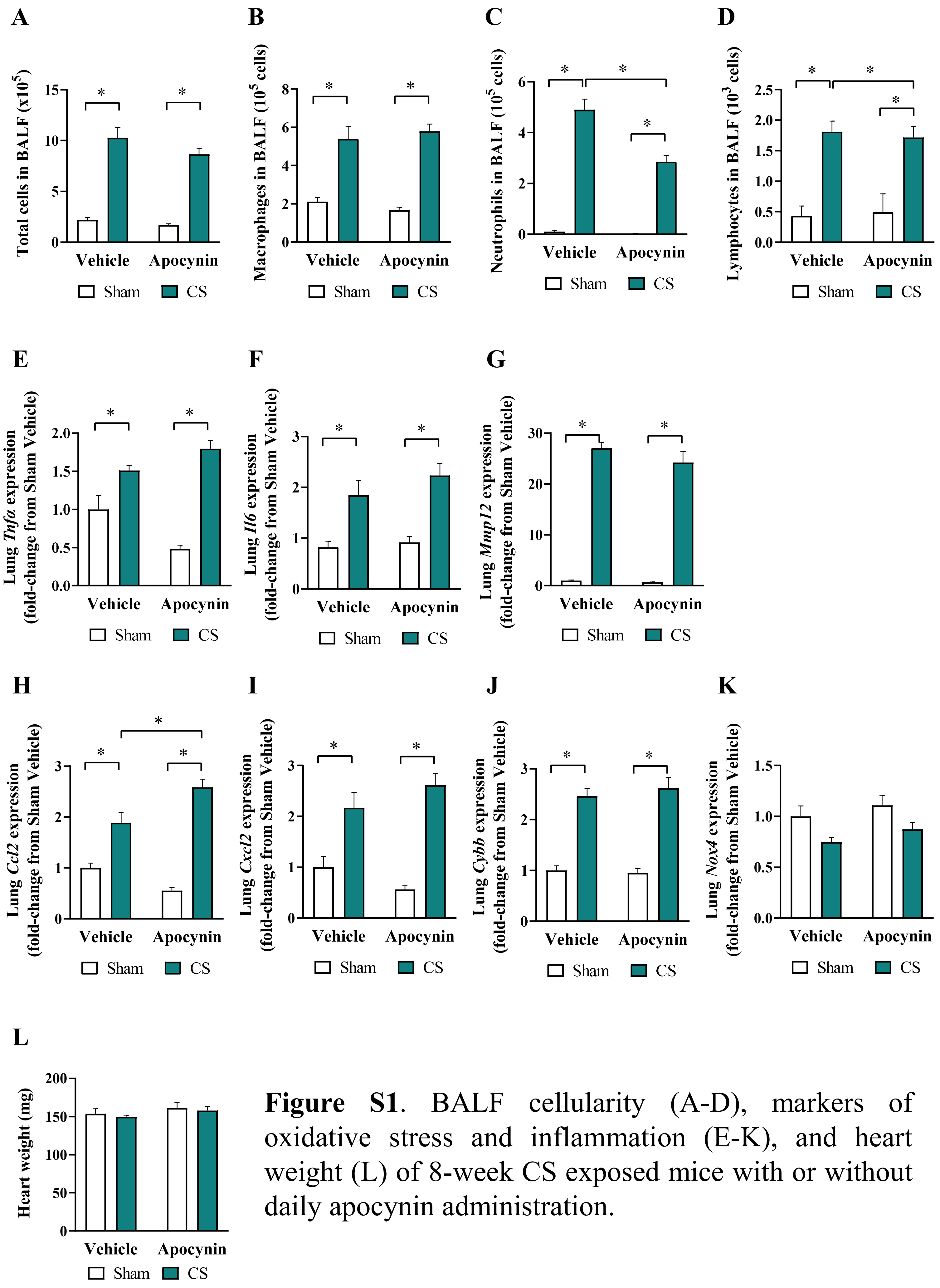

Figure S1. BALF cellularity (A-D), markers of oxidative stress and inflammation (E-K), and heart weight (L) of 8-week CS exposed mice with or without daily apocynin administration.

Figure S2: 24-week CS

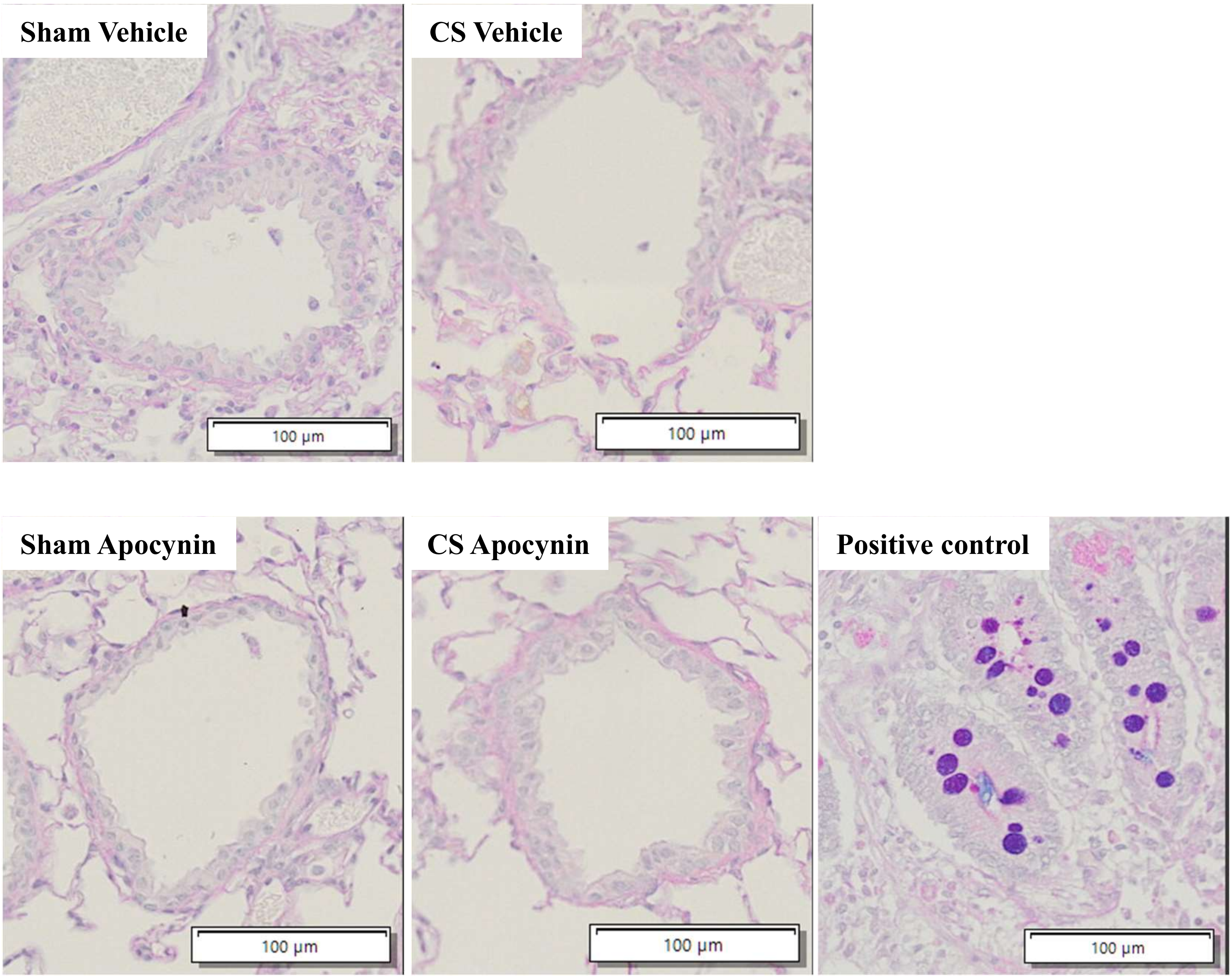

Figure S2. Representative images of lung sections stained with Alcian blue-periodic acid Schiff for mucus secretion. Kidney section was included as positive control.
